# Catalpol in Diabetes and its Complications: A Review of Pharmacology, Pharmacokinetics, and Safety

**DOI:** 10.3390/molecules24183302

**Published:** 2019-09-11

**Authors:** Ying Bai, Ruyuan Zhu, Yimiao Tian, Rui Li, Beibei Chen, Hao Zhang, Bingke Xia, Dandan Zhao, Fangfang Mo, Dongwei Zhang, Sihua Gao

**Affiliations:** 1 Diabetes Research Center, Traditional Chinese Medicine School, Beijing University of Chinese Medicine, Beijing 100029, China; bybucm@163.com (Y.B.); zhuruyuan7@163.com (R.Z.); tymtianyimiao@126.com (Y.T.); bucmlirui@163.com (R.L.); cbb8969@163.com (B.C.); zhangxiaohaoxyz@163.com (H.Z.); xiabk1230@163.com (B.X.); bucmzhaodandan@163.com (D.Z.); xiaofang.tcm@163.com (F.M.); 2 Sino-Canada Anti-Fibrosis Center, Traditional Chinese Medicine School, Beijing University of Chinese Medicine, Beijing 100029, China

**Keywords:** catalpol, diabetes, diabetic complications, pharmacology, pharmacokinetic, safety

## Abstract

This review aimed to provide a general view of catalpol in protection against diabetes and diabetic complications, as well as its pharmacokinetics and safety concerns. The following databases were consulted with the retrieval of more than 100 publications through June 2019: PubMed, Chinese National Knowledge Infrastructure, WanFang Data, and web of science. Catalpol exerts an anti-diabetic effect in different animal models with an oral dosage ranging from 2.5 to 200 mg/kg in rats and 10 to 200 mg/kg in mice. Besides, catalpol may prevent the development of diabetic complications in kidney, heart, central nervous system, and bone. The underlying mechanism may be associated with an inhibition of inflammation, oxidative stress, and apoptosis through modulation of various cellular signaling, such as AMPK/PI3K/Akt, PPAR/ACC, JNK/NF-κB, and AGE/RAGE/NOX4 signaling pathways, as well as PKCγ and Cav-1 expression. The pharmacokinetic profile reveals that catalpol could pass the blood-brain barrier and has a potential to be orally administrated. Taken together, catalpol is a well-tolerated natural compound with promising pharmacological actions in protection against diabetes and diabetic complications via multi-targets, offering a novel scaffold for the development of anti-diabetic drug candidate. Further prospective and well-designed clinical trials will shed light on the potential of clinical usage of catalpol.

## 1. Introduction

**Figure 1:**
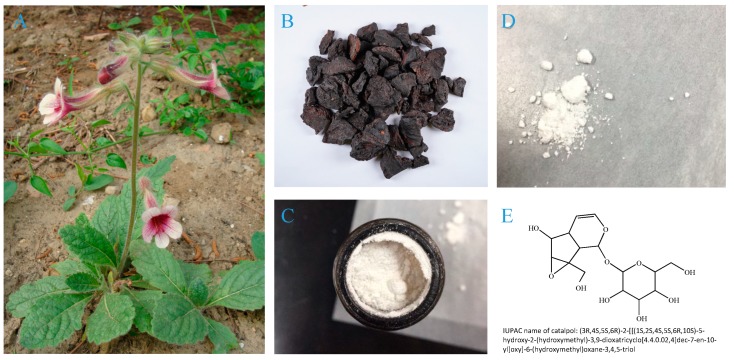
*Rehmannia glutinosa*, Catalpol, and its chemical structure. (**A**) *Rehmannia glutinosa*, (**B**) Shengdi (dry roots of *Rehmannia glutinosa*), (**C**,**D**) Crystallized powder of catalpol, (**E**) Chemical structure of catalpol. The International Union of Pure and Applied Chemistry (IUPAC) name of catalpol: (3*R*,4*S*,5*S*,6*R*)-2-[[(1*S*,2*S*,4*S*,5*S*,6*R*,10*S*)-5-hydroxy-2-(hydroxymethyl)-3,9-dioxatricyclo[4.4.0.02,4]dec-7-en-10-yl]oxy]-6-(hydroxymethyl)oxane-3,4,5-triol.

Catalpol is an iridoid glucoside widely found in plants belonging to several families of the order Lamiales, such as Plantaginaceae [1], Lamiaceae, and Bignoniaceae [2]. It was named after plants of genus *Catalpa,* in which it was first discovered in 1962 [3]. In 1971, Kitagawa Hiroshi first isolated catalpol from *Rehmannia glutinosa* (Figure 1) and proved its hyperglycemic effect [4]. With its origin distributed widely in the north and central part of China, *Rehmannia glutinosa* is one of the frequently used herbs in traditional Chinese medicine that was first recorded in Shen Nong’s Herbal Classic and had been clinically used for the management of diabetes for more than 1000 years [5,6]. Therefore, catalpol may assume the anti-diabetic action of *Rehmannia glutinosa* and has gained increasing attention for its potential contribution in controlling glycolipid metabolism and diabetic complications, making it a very promising scaffold for seeking new anti-diabetic drug candidate [7,8].

Diabetes mellitus (DM) [9] is a complex metabolic disorder caused by genetic and/or environmental factors, and it is characterized by sustained hyperglycemia due to deficient insulin secretion in islets or insulin resistance in the peripheral target organs [9,10]. DM has now become one of the major epidemics, and there will be approximately 629 million diabetic patients around the world by 2045 [11]. The current countermeasures for DM may run into bottlenecks for the potential side effects, which needs persisting search for new drug candidates [12,13]. For this purpose, we reviewed the current advances of catalpol in the management of diabetes and diabetic complications, its pharmacokinetic profiles, and safety concerns.

To accomplish this goal, we retrieved the following databases, including PubMed (www.pubmed.com), Chinese National Knowledge Infrastructure (www.cnki.net), WanFang Data (www.wanfangdata.com.cn/), and Web of Science (www.isiknowledge.com). The following words and/or phrases were used in various combinations, “catalpol”, “diabetes”, “insulin resistance”, “hyperglycemia”, “diabetic nephrology”, “diabetic cardiomyopathy”, “diabetic atherosclerosis”, “diabetic encephalopathy”, “peripheral neuropathy”, “diabetic osteoporosis”, “diabetic retinopathy”, “diabetic gastroparesis”, and “diabetic erectile dysfunction”. More than 100 scientific works of literature were consulted by June 2019.

## 2. Physicochemical Properties of Catalpol

In addition to mainly sourcing from *Radix Rehmanniae*, catalpol is also distributed in *Lancea tibetica* [14] and *Radix Scrophulariae* [15]. As a kind of iridoid glycoside with a polar structure, catalpol is soluble in water. However, catalpol is unstable under high temperature [16] and degrades rapidly at 100 °C (2–6 h) [17]. Besides, catalpol is less stable in the acidic environment compared to alkaline ones [18].

The content of catalpol depends on different processing and parts of plants [19]. It has been demonstrated that catalpol content is about 3–4% in fresh *Rehmannia glutinosa*, 1.79–2.28% in dry roots, and only around 0.27% after steaming. Moreover, except the roots of *Rehmannia glutinosa*, the stems and leaves of this plant are also rich in catalpol [20]. Xu et al. [21] found that the leaves of *Rehmannia glutinosa* contained around 3.81~24.51 mg/g of catalpol. This discovery provided a supplementary source for the extraction of catalpol.

## 3. Pharmacological Actions of Catalpol in the Management of Diabetes and Diabetic Complications

There were more than 30 papers to study the anti-diabetic efficacy of catalpol in the management of diabetes and its complications until June 2019. The animal models, route of administration, dose, treatment duration pertaining to the glucose-lowering effect of catalpol are summarized in Table 1.

### 3.1. The Hypoglycemia Effect of Catalpol in Diabetic Animal Models

Several diabetic rodent models have been used to study the effect of catalpol on diabetes and its complications. Of these, the most commonly used diabetic animal is Wistar rat, in which diabetic models are induced by intraperitoneal injection of streptozotocin (STZ) [33,34,35,36,37,38,42]. Besides, C57BL/6J mice (induced by STZ intraperitoneal injection) [23,25,26,27,30], *db*/*db* mice [28,29,30,39], KM mice [31], and KK-Ay mice [32] have also been employed to study the anti-diabetic effect of catalpol. Additionally, there is one study using alloxan-induced diabetic rabbits [41] to evaluate the effect of diabetic atherosclerosis (DA).

Regarding the intervention route, oral administration has been mostly applied in most of the studies, while intravenous and intraperitoneal injections have also been used in some investigations. The intervention dosages of catalpol are different among studies. For oral administration, the dosage ranges from 10–200 mg/kg·body weight (BW) in mice and 2.5–100 mg/kg·BW in rats (Table 1). The reason for this difference may be attributed to different animal models employed, treatment duration, and intervention routes. Furthermore, there is one study using catalpol-supplemented diet to evaluate the protective effects of catalpol on diabetic nephropathy [39].

Since Kitagawa firstly discovered the anti-hyperglycemia effect of catalpol in 1971 [4], emerging evidence has been accumulated to corroborate the hypoglycemic effect of catalpol. In an experiment conducted by Zhao et al., catalpol was orally administered at doses of 50, 100, 200 mg/kg·d for 2 weeks to alloxan-induced male diabetic KM mice (60 mg/kg, injection into the tail vein) [31]. The results revealed that catalpol could restore blood glucose and lipid profile and improved glucose tolerance. Also, catalpol has shown hypoglycemic effect in STZ-induced diabetic C57BL/6J mice [25,26]. In these experiments, catalpol was orally administrated for 4 weeks with the doses of 50, 100, 200 mg/kg.

Using *db*/*db* mice [28] and diabetic rats (induced by high-fat high-glucose (HFHG) and STZ) [34], catalpol (200 mg/kg vs. 2.5 and 5 mg/kg) has shown the ability to reduce the levels of fasting blood glucose (FBG) and fasting insulin. Interestingly, catalpol has been reported to ameliorate the hyperinsulinemia in *db*/*db* mice, and potentiate insulin expression in the islet of diabetic rats induced by HFHG diet plus STZ injection (15 mg/kg) [35].

In addition to oral administration, the following studies were conducted to evaluate the hypoglycemic effect of catalpol by intravenous injection. Huang et al. [33] found that the glucose-lowering effect of catalpol ranged from 8.53 ± 2.85% at the dose of 0.01 mg/kg to 24.33 ± 2.94% at 0.1 mg/kg in STZ-induced diabetic mice. Shieh. et al. [37] also claimed that catalpol dose-dependently (0.01, 0.05, and 0.1 mg/kg) exhibited a glucose-lowering effect in STZ-induced diabetic rats. The authors demonstrated that the increment of catalpol to 0.15 mg/kg did not exhibit further effect.

In short, catalpol demonstrates a glucose-lowering effect in the different diabetic rodent models, that can be administrated by gavage or by injection. The effective dosage of catalpol may vary from chemical-induced diabetic rats to genetic diabetic mice. Therefore, further investigations are needed to figure out the effective glucose-lowering dose using well-designed and strictly-controlled experiment, which will pave a scientific way for the potential clinical trial for this compound.

### 3.2. Catalpol and Liver

Currently, catalpol has been demonstrated to restore diabetic liver function through the following means. Firstly, catalpol is able to reduce hepatic gluconeogenesis and increase glycogen synthesis in high-fat diet (HFD) exposed C57BL/6J mice [23] and STZ-diabetic rats [37], as well as in *db*/*db* mice [30], by modulating the expressions of phosphoenolpyruvate carboxykinase (PEPCK), glucose-6-phosphatase, glycogen synthase kinase3β (GSK3β), and forkhead box protein O1, and activation of adenosine 5′-monophosphate-activated protein kinase (AMPK)/NADPH oxidase 4 (NOX4)/ phosphatidylinositol (−3) kinase (PI3K)/protein kinase B (Akt) signaling pathway. Using opioid μ-receptor knockout B6D2F1 mice, Shieh et al. [37] demonstrated that catalpol might decrease PEPCK expression through enhancing β-endorphin secretion via activation of opioid μ-receptors. Besides, catalpol has been demonstrated to activate the PI3K pathway in HL-7702 cells in response to insulin and dexamethasone stimulation [47]. Furthermore, using *db*/*db* mice, Liu et al. [29] demonstrated that catalpol could increase liver glucose metabolism genes expressions, such as insulin receptor substrate 1 (IRS1), isocitrate dehydrogenase 2, and glucose-6-phosphate dehydrogenase 2, and decrease gene expression of suppressor of cytokine signaling 3, an inhibitor from insulin signaling, in the liver [48].

Secondly, catalpol has the capacity of improving lipid metabolism in the liver. Bao et al. [30] demonstrated that catalpol was able to decrease serum adiponectin content and inhibit liver triglyceride and cholesterol synthesis through decreasing mRNA expression of acetyl-CoA carboxylase (ACC) and hydroxymethyl glutaric acid acyl CoA reductase (HMGCR) in *db*/*db* mice. Besides, catalpol also increases lipid metabolism genes expressions in the liver of *db*/*db* mice, such as acyl-CoA thioesterase 1, cytochrome P450-family 46-subfamily a-polypeptide 1, lipase, and fatty acid-binding protein 5 [29].

Thirdly, catalpol ameliorates diabetic liver through its antioxidant property. Using KK-Ay mice, Wang et al. [32] found that catalpol could improve serum lipids profile, and enhance liver antioxidant capacity, through upregulation of total-SOD (superoxide dismutase) and glutathione peroxidase (GSH-Px) activities and downregulation of malonaldehyde (MDA) content.

Lastly, catalpol has been evidenced to improve mitochondrial function through the promotion of mitochondrial fusion and inhibition of mitochondrial fission in the diabetic liver by regulating the expression of mitofusin-1 (MFN1), fission 1 (FIS1), and dynamin-related protein 1 (DRP1) [25]. The author also claimed that catalpol’s ability to decrease reactive oxygen species (ROS) might be related to the improvement of mitochondrial function. It is known that sustained high glucose may decrease MFN1 expression and increase FIS1 and DRP1 expressions, which consequently disturb normal mitochondrial fusion and fission, as well as redox homeostasis [49].

**Figure 2:**
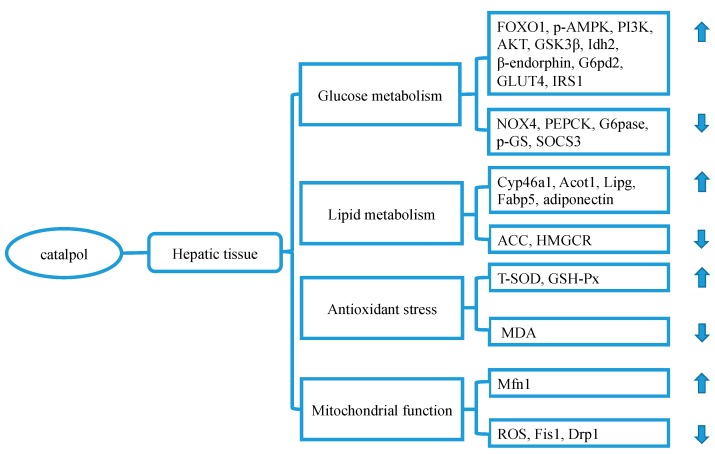
The relevant molecular targets of diabetic liver disorder modulated by catalpol. Catalpol is actively involved in improving glucose and lipid metabolism, ameliorating oxidative stress, and restoring mitochondrial function. Abbreviations: Acot1: acyl-CoA thioesterase 1, ACC: acetyl-CoA carboxylase, AMPK: adenosine 5‘-monophosphate-activated protein kinase, Akt: protein kinase B, Cyp46a1: cytochrome P450-family 46-subfamily a-polypeptide 1, Drp1: dynamin-related protein 1, Fabp5: fatty acid-binding protein 5, Fis1: fission 1, FOXO1: forkhead box protein O1, G6pase: Glucose-6-phosphatase, G6pd2: glucose-6-phosphate dehydrogenase 2, GLUT4: glucose transporter type 4, GSK3β: glycogen synthase kinase 3β, GSH-Px: glutathione peroxidase, HMGCR: hydroxymethyl glutaric acid acyl CoA, Idh2: isocitrate dehydrogenase 2, IRS1: insulin receptor substrate 1, Lipg: lipase, MDA: malonaldehyde, Mfn1: mitofusin-1, NOX4: NADPH oxidase 4, PEPCK: phosphoenolpyruvate carboxykinase, PI3K: phosphatidylinositol (−3) kinase, ROS: reactive oxygen species, SOCS3: suppressor of cytokine signaling 3, T-SOD: total superoxide dismutase.

In brief, catalpol exerts a beneficial effect on the diabetic liver through the promotion of glucose utilization and glycogen synthesis, as well as lipid metabolism (Figure 2). Also, catalpol can improve liver mitochondrial function. The mechanisms underlying these effects may be involved in regulating AMPK/NOX4/PI3K/Akt signaling pathway, and modulating multiple genes or proteins expressions, such as PEPCK, GSK3β, ACC, HMGCR, MFN1, FIS1, DRP1, and β-endorphin. Moreover, catalpol has been demonstrated to activate autophagy in carbon tetrachloride-induced liver fibrosis [50]. Given that autophagy insufficiency is actively involved in metabolic disorders [51], it is interesting to further investigate the effect of catalpol on autophagy in the diabetic liver.

### 3.3. Catalpol and Pancreatic Tissue

**Figure 3:**
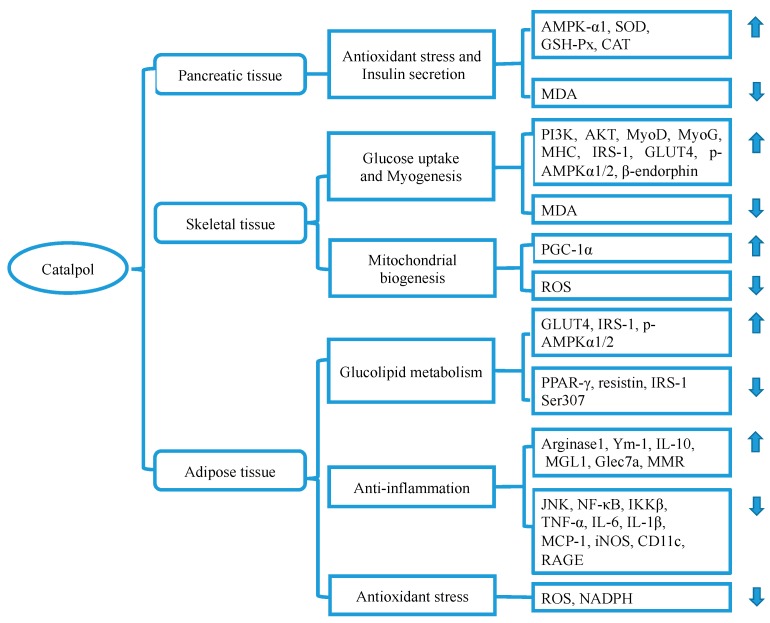
Catalpol regulates various genes and proteins of pancreatic, skeletal, and adipose tissue in the management of diabetes. Abbreviations: CAT: Catalase, IKKβ: inhibitory kappa B kinase, MHC: myosin heavy chain, JNK: c-Jun N-terminal kinase, MCP-1: Monocyte chemokine-1, MyoD: myogenic differentiation, MyoG: myogenin, NF-κB: nuclear factor-κB, PGC-1α: Peroxisome proliferator-activated receptor gamma coactivator 1-alpha, PPARγ: peroxisome proliferators-activated receptor-γ, RAGE: receptor for advanced glycation end product, TNF-α: Tumor necrosis factor-alpha.

Glucose homeostasis requires an appropriate amount of insulin produced by pancreatic β-cells. The accumulated glycolipid toxicity in the development of DM may gradually lead to β-cell dysfunction and exhaustion [52,53]. Catalpol may protect islet cells from fuel surfeit through the following ways [54,55] (Figure 3). Firstly, catalpol has been proved to boost insulin secretion by up-regulating the expression of AMPK-α1 in HFHG diet-induced diabetic rats [35]. Secondly, catalpol may play antioxidant effects in diabetic rats induced by STZ injection and HFHG diet exposure [36]. Catalpol may reduce inflammatory infiltration and fibrotic area through enhancement of superoxide dismutase (SOD), GSH-Px, and catalase (CAT) activity and attenuation of MDA concentration, which finally contributes to glycolipid homeostasis.

### 3.4. Catalpol in Adipose Tissue

Excessive lipid accumulation causes low-grade inflammation and subsequent insulin resistance [56,57]. Catalpol has been demonstrated to decrease M1 pro-inflammatory genes expressions, including tumor necrosis factor-α (TNF-α), interleukin-6 (IL)-6, IL-1β, monocyte chemotactic protein-1 (MCP-1), inducible NO synthase (iNOS), and CD11c, and increase M2 anti-inflammatory genes (arginase 1, Ym-1, IL-10, MGL1, Clec7a, and MMR) in HFD-induced obese mice. These effects have been mediated through suppression of c-Jun N-terminal kinase (JNK)/nuclear factor-κB (NF-κB) pathway [22].

Moreover, catalpol reduces advanced glycation end-products (AGEs)-induced inflammatory reaction and ROS over-generation in THP-1 cells [58]. In this context, catalpol (500 μM) reduces mRNA expressions of MCP-1 and TNF-α, and decreases protein expressions of iNOS and receptor for AGE (RAGE), as well as inhibits NADPH oxidase activity. This effect has been mediated through inhibition of activation of NF-κB and phosphorylation of extracellular signal-regulated kinase 1/2, p38, and JNK.

In addition, catalpol has also been demonstrated to improve glucose uptake and lipolysis by upregulating the expression of glucose transporter type 4 (GLUT4), IRS1, and p-AMPKα 1/2, as well as downregulating the expressions of peroxisome proliferators-activated receptor-γ (PPAR-γ), resistin, IRS1 Ser307 in 3T3-L1 adipocytes [59,60,61,62,63,64] and in the adipose tissue of *db*/*db* mice [30].

Taken together, the protective effect of catalpol on diabetic adipose tissue is a credit to its ability to alleviate inflammation, mitigating oxidation, and improving glycolipid metabolism through suppression of NF-κB activation and mitogen-activated protein kinase (MAPK) phosphorylation as well as AGEs stimulation (Figure 3). Considering the inhibitory effect of catalpol on NADPH oxidases, further investigation may be needed to evaluate which type of NADPH oxidases is involved in this process.

### 3.5. Catalpol in Skeletal Muscle Tissue

Skeletal muscle is the largest tissue responsible for glucose metabolic homeostasis. Diabetes may disrupt mitochondrial function in skeletal muscle, leading to an abnormality of myogenesis and impairment of energy metabolism [65,66]. Catalpol is evidenced to rescue the mitochondrial dysfunction and structure [26] through the induction of peroxisome proliferator-activated receptor-gamma coactivator 1-alpha (PGC-1α) expression in the skeletal muscle of HFD/STZ-induced diabetic mice. An increase in PGC-1α expression may contribute to mitochondrial biogenesis and respiratory capacity, as well as ROS emission [67], playing a beneficial role in diabetes management.

Besides, catalpol can promote myogenesis and improve skeletal muscle function, which contributes to glucose uptake and consequently leads to normalization of glycemia. Xu et al. [28] demonstrated that catalpol increased skeletal muscle grip strength and weight through upregulating the expression of myogenic differentiation (MyoD), myogenin (MyoG), and myosin heavy chain in *db*/*db* mice and C2C12 cell via the regulation of PI3K/Akt signaling pathway. Bao et al. [30] found that catalpol increased protein expression of p-AMPKα 1/2 and GLUT-4 expression in the skeletal muscle of *db*/*db* mice. Besides, Shieh et al. [37] found that the effect of catalpol on GLUT-4 expression might be associated with opioid μ-receptors-mediated β-endorphin secretion.

In a word, catalpol elaborates its anti-diabetic effect through the promotion of myogenesis and mitochondrial biogenesis in skeletal muscle (Figure 3). The mechanisms underlying these effects may be related to the regulation of PGC-1α expression and PI3K/Akt signaling pathway.

### 3.6. Catalpol and Diabetic Nephrology (DN)

Diabetic nephrology (DN) [68], one of the most prevalent and lethal diabetic complications, is now becoming the leading cause of end-stage renal disease in the world [69]. DN is recognized by albuminuria, increased serum creatinine, and low glomerular filtration rate [70], and usually caused by tubular basement and glomerular membrane thickening, extracellular matrix (ECM) accumulation, and progressive mesangial hypertrophy [71]. Catalpol may prevent the development of DN through the following means.

Firstly, catalpol has been demonstrated to improve lipid homeostasis in the diabetic kidney. Using *db*/*db* mice, Jiang et al. [39] claimed that catalpol was able to reduce 24-h urinary albumin excretion rate, serum creatinine, blood urea nitrogen, and restore renal physiology structure. These effects were associated with improvement of serum lipid markers and lipid metabolism genes (Scd2, Slc5a8, Ugt2b5, Acsm, Insig1, Gm20706, Map7, Gm-26160, Slc16a1, Hist1h1c, Mansc4, Ac-msd) in the kidney.

Secondly, catalpol has been evidenced to regulate insulin-like growth factor 1 (IGF-1) expression in protection against DN. It is generally believed that the induction of the IGF-1 triggers kidney hypertrophy and mesangial cell ECM accumulation, eventually accelerating the progress of DN [72,73] through the regulation of PI3K/Akt and MAPK pathways [74,75]. Meanwhile, the levels of IGF-1 are positively correlated with serum creatinine content [76]. Zhao et al. [40] found that catalpol could partially downregulate the expression of IGF-1 and Akt in the diabetic kidney, which may contribute to alleviating the pathological alterations of DN. In contrast, Yang et al. [24] demonstrated that catalpol preserved the kidney function by upregulating IGF-1 mRNA expression, IGF-1 receptor phosphorylation, and by suppressing the expression of Grb10, an adaptor protein that acts as the inhibitor of IGF-1. The inconsistent results between the two studies may be attributed to different diabetic animal models and different stages of DN.

**Figure 4:**
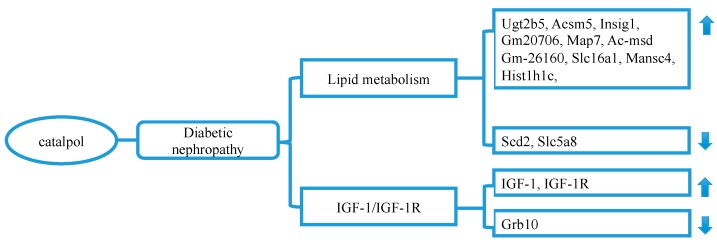
Catalpol alleviates renal damage through improving lipid metabolism, IGF-1 signaling, and inhibiting mesangial cell proliferation. Abbreviations: IGF: insulin-like growth factor, IGFR: insulin-like growth factor receptor.

In brief, catalpol may preserve diabetic renal function through the regulation of lipid metabolism and modulation of IGF-1 expression (Figure 4). However, how catalpol affects IGF-1 expression during the attack of DN still needs further investigation.

### 3.7. Catalpol and Diabetic Cardiopathy

Diabetic cardiopathy includes diabetic cardiomyopathy (DCM), DA, and cardiac autonomic neuropathy [77]. The main pathological manifestations of DCM are cardiomyocyte hypertrophy and myocardial fibrosis (MF) [78]. Sustained high-glucose may promote transforming growth factor-beta (TGF-β) overexpression, which leads to fibroblasts proliferation and ECM over-accumulation [79,80]. Catalpol has been proved to downregulate TGF-β1 expression in DCM mice, thereby protecting damaged myocardium, improving myocardial diastolic capacity, and delaying the development of MF [38]. Furthermore, catalpol could exert anti-apoptotic effect by inhibiting caspase-3 and BAX expressions and increasing Bcl-2 expression via regulating the nuclear paraspeckle assembly transcript 1 (NEAT1)/miR-140-5p/histone deacetylase 4 (HDAC4) signaling pathway in DCM mice and high glucose-stimulated mouse cardiomyocytes [27].

Diabetes may pose a higher risk of cardiovasculopathy [81,82]. Catalpol is evidenced to play a role in the management of cardiovasculopathy by its antioxidant and anti-inflammatory properties. Catalpol can increase Inhibitor of NF-κB α expression and decrease MDA, NF-κBp65, endothelin-1 expressions in human umbilical vein endothelial cells (HUVECs) in response to high glucose stimulation [83], and reduce IL-6 and TNF-α in endothelial cells [84]. Furthermore, catalpol inhibits neointimal proliferation and macrophage recruitment in DA rabbits [41]. These effects have been associated with an increase in the plasma activities of SOD and GSH-Px, and a decline in the plasma levels of MDA, AGEs, TNF-α, MCP-1, vascular cell adhesion molecule-1, and a reduction in the expressions of TGF-β1 and IV collagen in blood vessels. Also, catalpol regulates the levels of nitric oxide (NO) [85], lactate dehydrogenase (LDH), 8-iso-prostaglandin F2α, p22phox, and NOX4 [42] in diabetic vascular disorders.

**Figure 5:**
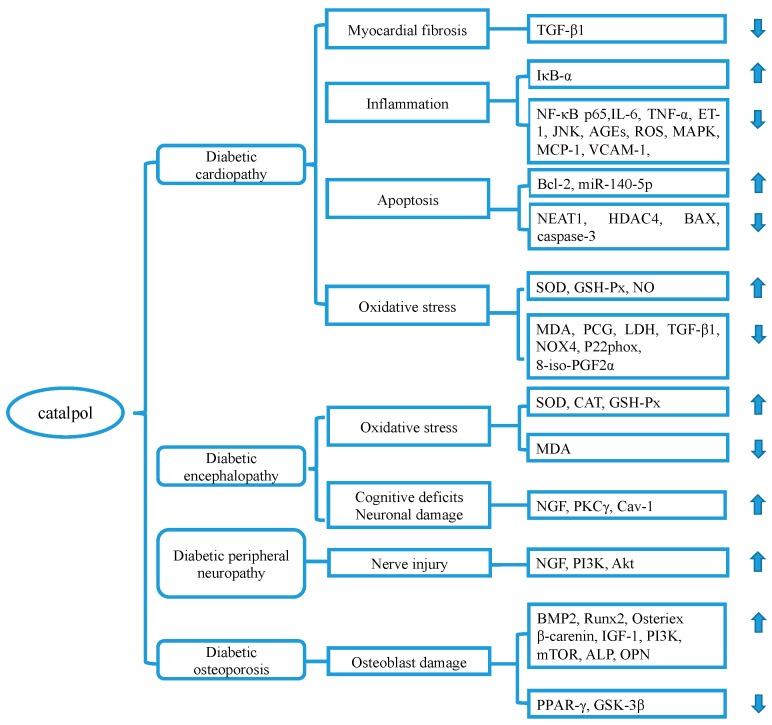
The relevant molecular targets of diabetic complications modulated by catalpol. Abbreviations: ALP: alkaline phosphatase, Cav-1: caveolin-1, ET-1: endothelin-1, HDAC4: histone deacetylase 4, IκB-α: NF-κB inhibitor-α, LDH: lactate dehydrogenase, MAPK: mitogen-activated protein kinase, mTOR: mammalian target of rapamycin, NEAT1: nuclear paraspeckle assembly transcript 1, NGF: nerve Growth Factor, NIP: neointimal proliferation, OPN: osteopontin, PCG: protein carbonyl groups, PKCγ: protein kinase C-gamma, TGF-β: transforming growth factor-beta, VCAM-1: vascular cell adhesion molecule-1.

In short, catalpol may exert cardiovascular protective effects through preventing myofibroblasts proliferation and inhibiting cardiomyocytes apoptosis by its anti-inflammatory and antioxidant characters (Figure 5). Also, several studies have proved that catalpol exerts a therapeutic effect on myocardial dysfunction and ischemia-reperfusion injury after myocardial infarction [86,87,88]. The mechanism underlying these effects may deserve further investigation.

### 3.8. Catalpol and Diabetic Encephalopathy (DE)

Diabetic encephalopathy (DE) refers to a chronic complication of DM characterized by electrophysiological, neurochemical, and structural abnormalities, cognitive impairment, and motor dysfunction [89]. Catalpol has been reported to prevent the development of DE through upregulation of GSH-Px, SOD, and CAT activity and downregulation of MDA content [46] (Figure 5). Besides, catalpol has been shown to ameliorate hippocampal injury and attenuate cognitive dysfunction through increasing nerve growth factor (NGF), protein kinase Cγ (PKCγ), and caveolin-1 (Cav-1) [43] expressions. It is known that PKCγ is closely related to spatial learning and synaptic plasticity [90]. Cav-1 is believed to be a biomarker in neurodegenerative diseases and take an active part in memory and learning [91].

Also, epidemiological data indicate that patients with DM are at higher risk of developing dementia or Alzheimer’s disease (AD) than the non-diabetic population [92]. Considering the substantial evidence that multiple genes and proteins (amyloid β-protein (Aβ)40, Aβ42 [93], choline acetyl-transferase [94], protein kinase C [95], adrenocorticotropic hormone, corticotropin releasing hormone [96]) expressions could be modulated by catalpol in protection against AD, these may also become new potential targets in the management of DE.

### 3.9. Catalpol and Diabetic Peripheral Neuropathy (DPN)

Diabetic peripheral neuropathy (DPN) is a kind of nerve-damaging diabetic disorder that affect peripheral nerves with up to 50% incidence among patients with diabetes [97]. Catalpol also plays an active role in the management of DPN (Figure 5). Both Zhou and Liu proved a therapeutic effect of catalpol in prevention against DPN [44,45]. In their studies, catalpol (5 mg/kg) was administrated to STZ-induced Sprague Dawley diabetic rats for 2 weeks. The results revealed that catalpol could improve nerve conduction velocity and preserve the histological structures of dorsal root ganglion and sciatic nerve. Also, the intervention with catalpol might lead to upregulation of IGF-1 and activation of PI3K/Akt signaling pathway.

### 3.10. Catalpol and Diabetic Osteoporosis

Diabetic osteoporosis is a chronic bone metabolic disease induced by hyperglycemia. It is characterized by compromised bone quality and destroyed bone microstructures, which lead to increased bone fragility and a higher risk of bone fracture [98]. Currently, there are a few studies on catalpol in the treatment of diabetic osteoporosis (Figure 5). Zhang studied the bone protective effect of six compounds from *Rehmanniae Radix* on MC3T3-E1 osteoblasts in response to high glucose stimulation [99]. The results showed that catalpol upregulated the expression of bone morphogenetic proteins 2, runt-related transcription factor 2 2, osteopontin, and osteriex, thereby increasing the activity of osteoblasts differentiation and bone matrix mineralization. The mechanisms underlying these effects may be associated with the inhibition of PPARγ and GSK-3β, and activation of IGF/β-catenin and PI3K/Akt/mammalian target of rapamycin (mTOR) signaling.

Besides, Lai et al. [100] studied the effect of catalpol on Th1/Th2 cells polarization in estrogen deficiency-induced bone loss. They demonstrated that bone mineral density was positively associated with the expression of Th2-specific transcription factor (GATA-3) and negatively associated with Th1-specific transcription factor (T-bet), indicating that catalpol might prevent bone loss by regulating Th1/Th2 paradigm. Given the intricate interacting network between osteoporosis and diabetes [101], it’s worthy of further investigating the effects and mechanisms of catalpol against diabetic osteoporosis.

## 4. Pharmacokinetics of Catalpol

The pharmacokinetic profile of catalpol has also been widely studied in normal and animal disease models. We have summarized the recent studies on pharmacokinetic profiles of catalpol, shown in Table 2.

Using LC/MS/MS method, Lu et al. [102] found that the pharmacokinetic parameters of catalpol (50 mg/kg by oral) were as the follows: t_1/2_ (1.212 ± 0.388 h), V_1/F_ (1.428 ± 0.681 L/kg), AUC_0−∞_ (69,520 ± 22,927 ng h/mL), MRT_0−∞_ (3.273 ± 0.365 h), T_max_ (1.333 ± 0.408 h), C_max_ (23,318 ± 10,468 ng/mL). Besides, the pharmacokinetics and bioavailability of catalpol at different doses after oral administration have also been studied in rats [103]. The results revealed that at 50, 100, and 200 mg/kg of catalpol, T_max_, t_1/2_, and C_max_ were 1.3 ± 0.4, 1.6 ± 0.8, 2.3 ± 0.8 h, and 1.8 ± 1.3, 1.7 ± 1.0, 2.6 ± 1.5 h, and 23 ± 10, 35 ± 14, 36 ± 9 μg/mL, respectively. The results indicated that catalpol was quickly absorbed after administration. The plasma concentration of catalpol was not increased in a dose-dependent manner. Also, the absolute bioavailability of catalpol was 66.7% in 50 mg/kg group, suggesting a potential of oral administration. However, one study compared the pharmacokinetic parameters of catalpol (50 mg/kg) by three administration routes [107]. The results showed that the bioavailability of intramuscular injection was 71.63%, and that of oral administration was 49.38%, suggesting that catalpol is unstable in the gastrointestinal tract.

Using high-performance liquid chromatography-atmospheric pressure chemical ionization-MS/MS method, Wang et al. [104] analyzed the distribution of catalpol in cerebrospinal fluid (CSF) and plasma of SD rats after intravenous injection with the dose of 6 mg/kg. The results demonstrated that catalpol could pass the blood-brain barrier with the AUC_CSF_/AUC_plasma_ of 5.8% and t_1/2_ of 1.5 h. Besides, the MRT_0–∞_ for CSF and plasma were 2.12 ± 1.0 h vs. 0.70 ± 0.20 h, respectively. These results may provide a material basis for catalpol in the treatment of DN.

Several groups compared the pharmacokinetic data of catalpol under different animal diseases’ models. Feng et al. [105] found that the AUC_(0-t)_ in normal and diabetic (STZ; 55 mg/kg) rats were 92.4 ± 38.2 μg/mL·L and 214.1 ± 79.03 μg/mL·L, respectively. Meanwhile, the C_max_, T_max_, t_1/2_ were 11.82 ± 6.79 μg/mL vs. 37.41 ± 13.01 μg/mL, 3.1 ± 1.7 h vs. 2.4 ± 1.1 h, and 4.0 ± 1.0 h vs. 11.6 ± 4.2 h, respectively. Liu et al. [107] also studied that pharmacokinetic profile of catalpol in alloxan-induced diabetic rats. They found that the effective dose (hypoglycemic and lipid-lowering effect) of catalpol for oral administration was 200 mg/kg, while that for intravenous injection were 5, 10, 20 mg/kg with significant dose-dependency. Furthermore, Zhao et al. [106] found that the pharmacokinetic parameters for catalpol (8.0 mL/kg) revealed that AUC_(0-t)_ (439.71 ± 42.34 μg/min·mL), C_max_ (7.94 ± 1.06 μg/mL), T_max_ (60.50 ± 15.00 min), and t_1/2_ (84.00 ± 13.42 min) in normal rats were different from those (1627.83 ± 241.40 μg/min·mL, 2.14 ± 0.13 μg/mL, 54.00 ± 39.13 min, and 118.28 ± 3 9.13 min) in chronic kidney disease rats.

The reasons for pharmacokinetic parameters alterations of catalpol in different conditions are complicated. The gastrointestinal micro-environment, liver microsomal enzymes (CYP) [107], drug transporters (P-glycoprotein), and binding of plasma protein (glucuronidation and sulfation) all interfere with the absorption, distribution, metabolism, and excretion of catalpol. Indeed, Chen et al. [108] found that catalpol was metabolized into one isomer and five transformation products in rat plasma after oral administration, including catalpol isomer, methylation, hydrogenation, glycosylation, deoxyhydroxylation, and deoxyhydroxylation methylation.

## 5. Safety Concern of Catalpol

So far, it is demonstrated that catalpol is rather safe for rodent animals with no obvious side effects. Dong et al. [109] studied acute toxicity of catalpol in Institute of Cancer Research mice (maximum dose of 1000 mg/kg) and found no obvious toxic symptoms occurred after oral administration. The eating and activities of all mice appeared normal. Jiang et al. [110] conducted acute toxicity experiment in KM mice and long-term toxicity experiment in Wistar rats. They found that median lethal dose of catalpol was 206.5 mg/kg by intraperitoneal injection in mice. Also, no toxic changes were shown in biochemical indicators and physiological structures of organs after long-term intravenously administration of catalpol, indicating that this compound has no obvious toxic and side effects after long-term application.

Besides, Fei et al. [111] performed a preliminary experiment to evaluate catalpol’s safety in the treatment of patients with colon cancer. For 12 consecutive weeks, 10 mg/kg of catalpol was injected intraperitoneally, twice a day. The results showed that only mild non-fatal adverse events were found, such as nausea, vomiting, gastrointestinal ulcers, and constipation. In a word, current studies indicate that catalpol is well-tolerated with no-toxicity in rodent animals and patients with colon cancer.

## 6. Conclusions and Perspectives

**Figure 6:**
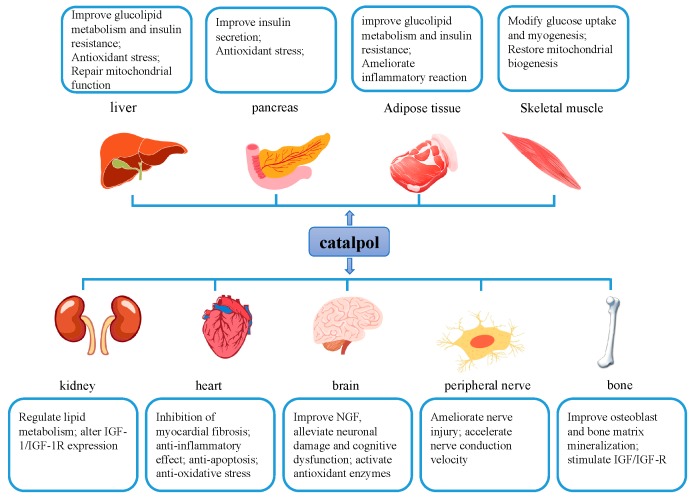
Catalpol reduces diabetic complications and disorders in different systems and/or tissues, including liver, pancreas, adipose tissue, skeletal muscle, bone, kidney, cardiovascular system, and central and peripheral nervous system.

Recent studies have provided scientific evidence for catalpol in protection against diabetes and its complications (Figure 6). The mechanism underlying these effects may be associated with the regulation of AMPK/PI3K/Akt, PPAR/ACC, JNK/NF-κB, AGE/RAGE/NOX4, and NEAT1/miR-140-5p/HDAC4 signaling pathways, as well as PKCγ and Cav-1 expressions. Also, the properties of anti-inflammation and antioxidant of catalpol contribute to its anti-diabetic effect. Moreover, the dosage and treatment duration from different investigations show wide discrepancies, which may interfere with the explanation of the pharmacological actions.

Catalpol is soluble in water and unstable under high temperature and acid environment. The pharmacokinetic profile reveals that catalpol is quickly absorbed after oral administration and could pass the blood-brain barrier, which makes this compound to have a possibility to treat the patients with DE. Besides, the glucose-lowering effective dose of catalpol for oral administration is 200 mg/kg, while that for intravenous injection is 5 mg/kg, which may guide the further investigation. However, there are inconsistent results regarding the bioavailability of catalpol after oral administration, that deserves further investigations.

As a well-tolerated and no safety concern compound, catalpol may have a chance to be used in diabetic clinical trials. However, the translation from the available data and evidence from the preclinical studies into clinical trial still needs a long way to go. Given that catalpol has already been clinically applied in treating tumors, the compound may provide a new option to the management of patients with DM and its complications. In this regard, a properly-designed, double-blind, and multi-center clinical study is warranted, which may provide strong evidence for its clinical application.

There are some limitations to the current review. Firstly, due to restrictions on accessibility, we only consulted articles in the selected Chinese and English databases. We did not review the research advances of catalpol in other languages, and Scopus and ScienceDirect databases. Secondly, the quality of the included studies varied. The reliability and reproduction of the results are not guaranteed. All these may undermine the conclusion of the current review. However, this is, to our knowledge, the first comprehensive review on the role of catalpol in diabetes and its complications.

In conclusion, catalpol is a safe, non-toxic natural compound and demonstrates a prominent role in the management of diabetes and its complications in preclinical studies. Its brisk pharmaceutical activity in various tissues and organs makes it a promising anti-diabetic drug candidate that deserves further investigations.

## Figures and Tables

**Table 1:** Diabetic animal models used in studying the glucose-lowering effects of catalpol.

Animal Model	Route of Administration	Dose of Catalpol	Treatment Duration	Reference
C57BL/6J + HFD	oral	100 mg/kg	4w	[22]
C57BL6/J + HFD/STZ (40 mg/kg)	oral	100, 200 mg/kg	4w	[23]
C57BL/6 + STZ (180 mg/kg)	i.p.	10 mg/kg	2w	[24]
C57BL/6J + HFD/STZ (85 mg/kg)	oral	50, 100, 200 mg/kg	4w	[25,26]
C57BL6/J + HFHG/STZ (100 mg/kg)	oral	10 mg/kg	12w	[27]
*db*/*db* mice	oral	200 mg/kg	8w	[28]
*db*/*db* mice	oral	20, 50, 100, 200 mg/kg	8w	[29]
*db*/*db* mice	oral	40, 80, 120 mg/kg	4w	[30]
KM + ALX (60 mg/kg)	oral	50, 100, 200 mg/kg	2w	[31]
KK-Ay mice	oral	200 mg/kg	8w	[32]
Wistar + STZ (60 mg/kg)	i.v.	0.1 mg/kg	/	[33]
Wistar + STZ (15 mg/kg)	oral	2.5, 5, 10 mg/kg	12w	[34,35]
Wistar + STZ (30 mg/kg)	i.v.	5, 10, 20, 50 mg/kg	2w + 2w	[36]
Wistar + STZ (65 mg/kg) BDF1/opioid receptor gene knockout mice + STZ (50 mg/kg)	i.v.	0.1 mg/kg	3d	[37]
Wistar + STZ (15 mg/kg)	oral	2.5, 5, 10 mg/kg	12w	[38]
*db*/*db* mice	chow diet supplemented with catalpol	1 g/kg	16w	[39]
SD + STZ (50 mg/kg)	i.p.	5 mg/kg	2w	[40]
Rabbit + ALX (100 mg/kg)	oral	5 mg/kg	12w	[41]
Wistar + STZ (30 mg/kg)	oral	10, 50, 100 mg/kg	6w	[42]
SD + STZ (50 mg/kg)	i.p.	5 mg/kg	2w	[43,44,45]
SD + STZ (65 mg/kg)	oral	10, 50, 100 mg/kg	6w	[46]

Note: i.v.: intravenous injection, i.p.: intraperitoneal injection; HFD: high-fat diet; HFHG: high-fat high-glucose diet, STZ: streptozotocin, KM: Kunming mice, ALX: Alloxan.

**Table 2:** Pharmacokinetics of Catalpol.

Experimental Animal	Dosage and Methods	Pharmacokinetic Parameters							Reference
		t_1/2_/h	AUC/(mg·h·L^−^^1^)	MRT/h	C_max_/(mg/L)	T_max_/h	V_z/F_/(L·kg^−^^1^)	F%	
Healthy male SD rats	50 mg/kg, gavage	1.39 ± 0.22	95.23 ± 10.15	3.23 ± 0.37	24.83 ± 0.58	1.66 ± 0.58	1.17 ± 0.16	49.38 ± 10.54	[18]
Healthy male SD rats	50 mg/kg, i.p.	0.84 ± 0.41	150.23 ± 20.87	1.62 ± 0.20	80.43 ± 5.59	0.25 ± 0.1	0.40 ± 0.20	71.62 ± 10.28	[18]
Healthy male SD rats	50 mg/kg, i.v.	0.68 ± 0.24	195.79 ± 20.51	1.71 ± 0.29	110.82 ± 4.10	/	0.57 ± 0.19	/	[18]
Healthy male and female Wistar rats	50 mg/kg, gavage	1.21 ± 0.39	69.52 ± 22.93	3.27 ± 0.37	23.32 ± 10.47	1.33 ± 0.41	1.43 ± 0.68	/	[102]
Healthy male and female Wistar rats	50 mg/kg, gavage	1.8 ± 1.3	70 ± 23	3.3 ± 0.4	23 ± 10	1.3 ± 0.4	3.6 ± 1.4	66.70	[103]
Healthy male and female Wistar rats	100 mg/kg, gavage	1.7 ± 1.0	118 ± 27	3.4 ± 0.5	35 ± 14	36 ± 9	3.7 ± 3.1	/	[103]
Healthy male and female Wistar rats	200 mg/kg, gavage	2.6 ± 1.5	217 ± 49	6.8 ± 1.2	36 ± 9	2.3 ± 0.8	14.7±8.8	/	[103]
Healthy male and female Wistar rats	50 mg/kg, i.v.	1.9 ± 1.5	104 ± 11	1.9 ± 0.4	/	/	4.1 ± 2.0	/	[103]
Healthy male SD rats	6 mg/kg, i.v. (in CSF)	1.52 ± 0.74	0.67 ± 0.11	2.12 ± 1.0	0.68 ± 0.20	/	/	/	[104]
Healthy male SD rats	6 mg/kg, i.v. (in plasma)	0.71 ± 0.23	11.53 ± 1.64	0.70 ± 0.20	23.61 ± 0.91	/	/	/	[104]
Healthy male SD rats	37.2 mg/kg, gavage	4.0 ± 1.0	93.7 ± 38.6	/	11.82 ± 6.79	3.1 ± 1.7	/	/	[105]
STZ (55 mg/kg) induced diabetic rats	37.3 mg/kg, gavage	11.6 ± 4.2	220.2 ± 79.6	/	37.41 ± 13.01	2.4 ± 1.1	/	/	[106]
Healthy male SD rats	8.0 mg/kg, gavage	1.4 ± 0.2	7.41 ± 0.68	/	2.14 ± 0.13	1.01 ± 0.25	/	/	[106]
Doxorubicin (5.0 mg/kg) induced CKD rats	8.0 mg/kg, gavage	2.0 ± 0.7	27.64 ± 4.20	/	7.94 ± 1.06	0.90 ± 0.14	/	/	[106]

Note: i.v.: intravenous injection. i.p.: intraperitoneal injection. AUC: area under curve, CSF: cerebrospinal fluid.
